# Nonparetic Knee Extensor Strength Is the Determinant of Exercise Capacity of Community-Dwelling Stroke Survivors

**DOI:** 10.1155/2014/769875

**Published:** 2014-08-14

**Authors:** Wei-Te Wang, Ling-Tzu Huang, Ya-Hui Chou, Ta-Sen Wei, Chung-Che Lin

**Affiliations:** Department of Physical Medicine and Rehabilitation, Changhua Christian Hospital, No. 135 Nan-Hsiao Street, Changhua 500, Taiwan

## Abstract

*Objective.* To investigate the relationship among walking speed, exercise capacity, and leg strength in community dwelling stroke subjects and to evaluate which one was the leading determinant factor of them.* Design.* This is a descriptive, cross-sectional study. Thirty-five chronic stroke patients who were able to walk independently in their community were enrolled. Walking speed was evaluated by using the 12-meter walking test. A maximal exercise test was used to determine the stroke subjects' exercise capacity. Knee extensor strength, measured as isokinetic torque, was assessed by isokinetic dynamometer. * Results.* The main walking speed of our subjects was 0.52 m/s. Peak oxygen uptake (VO_2_ peak) was 1.21 ± 0.43 L/min. Knee extensor strength, no matter whether paretic or nonparetic side, was significantly correlated to 12-meter walking speed and exercise capacity. Linear regression also showed the strength of the affected knee extensor was the determinant of walking speed and that of the nonparetic knee extensor was the determinant of exercise capacity in community dwelling stroke subjects. * Conclusions.* Walking speed and peak oxygen uptake were markedly decreased after stroke. Knee extensor strength of nonparetic leg was the most important determinant of exercise capacity of the community-dwelling stroke subjects. Knee extensor strengthening should be emphasized to help stroke patient to achieve optimal community living.

## 1. Introduction

Stroke is a leading cause of death and disability [1]. Walking ability is a priority of functional recovery from stroke. About two-thirds of patients can regain an independent walking ability after stroke, but only 7% of patients can regain independent community ambulation [2, 3]. Reduced muscle strength and exercise capacity are well-documented impairments in the stroke patient and also important factors that limit walking ability of stroke patients [4, 5].

After stroke, walking capacity is reduced due to motor control and muscle strength impairments [6]. Hemiparetic patients exhibited an asymmetrical gait pattern and compensative walking performance which cause speed reduction [7]. Walking speed of stroke patients was only approximately half that of an age-matched healthy group [4]. In the study of walking performance of the stroke subjects, walking speed has been reported as the most important predictor of unlimited community ambulation [8].

Hemiplegic gait requires the expenditure of a large amount of extra energy, and what is worse, the stroke patients often do not have enough exercise capacity to overcome [4, 9, 10]. The exercise capacity of stroke patients is only 50% to 60% that of healthy subjects [4, 5]. Like walking speed, impaired exercise capacity affects walking performance and is also an important factor to determine the return of stroke subjects to the community environment [4].

Structural and lean mass abnormality of hemiparetic leg muscles were observed after stroke [11]. They lead to not only muscle weakness but also muscle oxygen uptake decrease. Lower limb muscle strength, especially strength during knee extension, has been found to have high correlation with walking ability after stroke in previous studies [12, 13]. Similarly, the correlation between low limb muscle strength and exercise capacity was well studied in many clinical groups, like chronic obstructive pulmonary disease (COPD) and heart disease [14, 15]. However, to our knowledge, there was no study directly mentioned about this relationship in stroke patient. Only one study found that reduction of large leg muscle mass has a negative relationship with exercise capacity [16].

In stroke rehabilitation, exercise to strengthen muscles for walking and endurance training for increasing exercise capacity both are important regimens to help stroke patients to achieve walking independently in their community. Like endurance training, strengthening exercise can also elevate exercise capacity indirectly by increasing muscle oxygen extract. The aim of this study was to explore the chronic stroke patients who have unlimited community ambulatory ability and to measure the relationships among knee extensor strength, walking speed, and exercise capacity. Understanding their relationships and finding out primary determinants can help us to choose more efficient training regimens in present stroke rehabilitation program.

## 2. Methods

### 2.1. Subjects

Thirty-five chronic stroke patients, aged 20 to 80 years, were recruited for this study. All stroke subjects had undergone outpatient rehabilitation. All had a diagnosis of hemisphere stroke confirmed clinically through computed tomography (CT) scans or magnetic resonance imaging (MRI). Inclusion criteria were (1) infarctic or hemorrhagic hemiparetic stroke and (2) ability to walk independently in their community with or without a device. Exclusion criteria included (1) stroke patient who was intolerant to exercise testing, (2) some conditions or comorbidities that were a contraindication to the American College of Sports Medicine (ACSM) guideline, (3) musculoskeletal restrictions that would impede the cycle ergometer or isokinetic test, and (4) refusal to sign informed consent. This study was approved by the Institutional Review Board of the Changhua Christian Hospital (Protocol no. :  081023), and all subjects signed informed consent before being enrolled in the study.

### 2.2. Walking Speed

The walking speed test was performed in a gait analysis laboratory. Subjects were instructed to walk in a straight line at a comfortable pace without verbal encouragement. Walking speed was calculated from the time taken to walk 12 meters in a straight line. The 12-meter walk was repeated twice, with a 2-minute rest between tests. The time needed to walk the last 6 meters was also recorded. The best time was recorded and represented the stroke subject's walking speed. It was thought that during the last 6-meter walk, the subjects would be able to offset the initial resistance and enter the stage of stable walking. Five subjects used an orthosis or waking aid in the test.

### 2.3. Strength

Measurements of muscle strength were performed using the Biodex (Biodex, Corp., Shirley, NY) isokinetic dynamometer. The patient was seated comfortably with his back against a backrest and strapped at a hip angle of 90 degrees. The leg was attached to the lever arm of the dynamometer. All participants had a practice session before the actual testing to reduce the learning effect. The nonparetic leg of every patient was evaluated first, followed by the paretic leg. The test was repeated three times and the maximal torque was recorded as the subject's strength.

Previous study has reported high angular velocity cannot be performed by hemiplegic subjects because of increasing effect of velocity-dependent spasticity [17]. Therefore, we adopt low angular velocities, 60 and 90 degrees per second (degrees/s), to measure concentric isokinetic knee extension strength of both legs. The isokinetic dynamometer is a more accurate measurement of muscle strength and many studies have offered evidence of the ability to achieve an objective and reliable evaluation of the strength of patients with stroke [18, 19].

### 2.4. Exercise Capacity

Exercise capacity or aerobic capacity is defined as the maximum amount of oxygen the body can use during exercise and can represent cardiopulmonary fitness. It can be quantified clinically by measurement of peak oxygen uptake (VO_2_ peak), ventilatory threshold, minute ventilation (VE), maximal heart rate (HRmax), and other parameters of gas analysis in the maximal tolerance test. The maximal tolerance test, conducted on a cycle ergometer (Vision, Recumbent Bike), was used to determine the subjects' exercise capacity. An incremental protocol was performed. In warm-up period, intensity was set of level I resistance with the pedal rate of 40 rpm (about 23 W) for 3 minutes. In the beginning of test period, the pedal rate raised to 50 rpm and subjects were asked to keep at this rate throughout the test period (The initial intensity of the test period was about 31 W). Then the intensity increased by 2 resistance levels every 2 minutes during the test period. The intensity changed every two minutes in the order of 40 W, 51 W, 65 W, 81 W, 99 W, and 118 W. Gas exchange was recorded by measuring the expired gas flow with gas meters using a facial mask (COSMED, K4b2 system). Heart rate and blood pressure were measured once every minute during the test. VO_2_ peak, maximal heart rate, and maximal ventilation were measured. VO_2_ peak was used instead of VO_2_max to measure exercise capacity because stroke individuals usually have difficulty meeting a vigorous intensity challenge. The ventilatory threshold occurred very early in the exercise test and was therefore difficult to interpret. The individual was encouraged to continue the test for as long as possible. All the tests on the cycle ergometer were performed and supervised by a physical therapist.

### 2.5. Date Analysis

Descriptive statistics were expressed as mean and standard deviation. For 2 or more measures comparison, the significant levels were calculated by an independent *t*-test and a one-way analysis of variant test (ANOVA). A Pearson's correlation analysis was used to assess the relationship between walking speed, knee extensor strength, and exercise capacity (VO_2_ peak). Measures significantly correlated with walking speed and exercise capacity were used as independent variables in univariate linear regression. Linear regression analyses were further used to test whether a model incorporating these independent variables (like muscle torque from knee extensors, height, weight, etc.) could predict or determine walking speed or exercise capacity. All analyses were performed using SPSS, version 18.0. A correlation was considered significant with a *P* value of <0.05.

## 3. Results

A total of thirty-five chronic stroke patients were enrolled in the study. Their average age was 57.10 ± 12.14 years, and they were tested at a median of 1.84 years after stroke. The demographic and medical characteristics of the population are presented in Table 1. The difference in the mean between the *β*-blocker user and the nonuser or the smoker and the nonsmoker or the regular and the irregular exercise habits groups for the VO_2_ peak, VE, and HRmax was not statistically significant.

The main walking speed of the subjects was 0.52 ± 0.28 m/s and the last 6-meter walking velocity was faster than that of the first 6 meters (Table 2, *P* < 0.00). Average VO_2_ peak was 1.21 ± 0.43 L/min. The knee extensor torques, no matter the 60-degree or 90-degree, were lower for the paretic limb than for the nonparetic limb (*P* < 0.00). The 60-degree torques were higher than the 90-degree torques (*P* < 0.00). The average torque values of the paretic limb were about 66% those of the nonparetic limb.

The correlations with walking speed and VO_2_ peak were presented in Table 3. The 90-degree torques had highest coefficient no matter with walking speed or VO_2_ peak. The 12-meter walking speed was mostly related to 90-degree torques for knee extensors of paretic limbs (Table 3, *r* = 0.662, *P* < 0.00). The VO_2_ peak was mostly related to 90-degree torques of nonparetic knee extensors (Table 3, *r* = 0.606, *P* < 0.00). There were also high correlations between 60-degree and 90-degree torques. Therefore, the 90-degree torques were chosen to represent muscle strength of leg.

Given this relationship, multiple linear regression models were performed to determine which variables can predict walking speed and exercise capacity. The independent variables in these models included age, BMI, duration, FIM, and 90-degree torques of paretic and nonparetic knee extensors. When models were used to predict peak oxygen uptake, walking speed was included as a covariate (Table 4). The 90-degree torques for knee extensors of paretic limbs were the only independent predictor for walking speed (*β* = 0.621, *P* < 0.05) and accounted for 49.7% of the variance. The 90-degree torques for knee extensors of nonparetic limbs were the only factor to predict peak oxygen uptake. (*β* = 0.559, *P* < 0.05) and accounted for 42.2% of the variance.

## 4. Discussion

We observed a significant correlation between knee extensor strength, VO_2_ peak, and 12-meter walking speed of the community-dwelling stroke patients. The strength of the paretic knee extensor was the most important determinant of walking speed. Moreover, the exercise capacity was mostly affected by the nonparetic knee extensor strength.

Isokinetic assessments have shown high reliability in test and are objective evaluations of strength in patients with stroke [18, 20]. As we understand, rapid knee extension, in deceleration and heel contact, plays an important role and would be a determinant of step length and walking speed [21]. Many studies have investigated the relationship between knee extensor strength of the paretic leg and gait performance in stroke [13, 21–24], and all showed positive results. The result of this study coincided with previous findings [21, 24] that both 60- and 90-degree torques of the paretic knee extensors showed a moderate correlation to walking speed. However, for the strength of nonparetic limb, conflict results of correlation with walking ability have been reported [13, 21–24]. Although there was a low correlation among the strength of nonparetic limb and walking speed in this study, the result of multiple linear regression models, given these variables in, showed only 90-degree torque of the paretic leg was the strongest determinant of walking speed.

Self-selected walking speed had a strong correlation to VO_2_ peak in previous studies of stroke [16, 25]. However, our results indicated that walking speed had a lower correlation (*r* = 0.465, *P* = 0.005) to VO_2_ peak. Possible explanations were that, firstly, the subjects in the walking test of this study were not hastened. Previous studies showed the walking speed is about 1.5 m/s for healthy adults and was about 0.63~1.02 m/s for hemiplegic stroke subjects [4, 16]. The main walking speed of the subjects in this study was 0.52 m/s, which was 18%~48% lower than that of patients in previous studies and only about one-third that of a healthy group. Secondly, in the present study, there were five subjects still had to use an orthosis or walking aid in their daily life. Their walking speed was much slower, but their exercise capacity and leg strength had no difference when compared with other subjects who did not use aid devices (this additional statistical analysis is not presented in the paper). This low walking speed was probably not influenced by poor recovery of leg strength or exercise capacity; it might result from other facts, like balance, spasticity, or sensory loss. Therefore, these reasons may explain partially for the lower correlation between VO_2_ peak and walking speed. Walking at a comfortable pace rendered the tested values too slow to represent the optimal performance of the stroke subjects, even though this may have reflected their ordinary walking pattern.

The relationship between strength and exercise capacity was identified in many clinical groups. In studies of above issue, leg strength was most frequently raised for discussion. In a cross-section study of COPD population, quadriceps strength showed significant correlation to exercise capacity, which is evaluated by 6-minute walk test [15]. In postcoronary artery bypass graft surgery (CABG) patients, maximal quadriceps isometric strength was a good predictor of exercise capacity [26]. Quadriceps strength and peak oxygen consumption also showed significant high correlation in heart failure population [27]. However, in chronic stroke group, there were few studies for relationship between leg strength and exercise capacity. There was one study which discussed lean mass of both thighs with exercise capacity of the chronic stroke patients [16].

The stroke results in muscle weakness, balance disorders, spasticity, and substantial muscle atrophy that are accompanied with lean muscle mass reduction. In the structural changes of muscle in the poststroke patients, there is an evidence that the proportion of intramuscular fat [28] and fast myosin heavy chain increased [29] in paretic limbs. All these factors above may contribute to the impaired oxygen utilization of muscle, thereby reducing the exercise capacity of stroke patients. On the other hand, in the study by Ryan et al. [16], there was also a gain in lean muscle mass in the nonparetic leg because the patients were more dependent on it in locomotion after stroke. In above study, both paretic and nonparetic legs had a similar negative correlation to VO_2_ peak and no evidence showed which one was the more prominent determinant. In our study, even though the knee extensor strength of both paretic and nonparetic legs had a similar moderate correlation to VO_2_ peak, however, in regression model, we found that the knee extensor strength of the nonparetic limb was the most powerful determinant of the exercise capacity of ambulatory community-dwelling stroke survivors.

The limitations of this study include, firstly, that cycle ergometer was used to assess the exercise capacity of the stroke patients. The VO_2_ peak values during cycle ergometer testing were lower than those with the treadmill test, and the test may terminate prematurely before maximal effort due to leg fatigue. We chose cycle ergometer because it was safe for hemiplegic patients and accidental falls could be prevented. In addition, impaired walking ability leads to difficulty for stroke patients to walk effectively on a treadmill. The second limitation was that only knee extensor was tested and used to represent the stroke subject's leg strength. Because of the variations in the impairment of stroke subjects, there was a major problem that muscles in one subject recovered incongruously. Some paretic limbs were too weak to perform isokinetic dynamometer testing. Knee extensor strength has been well studied and has been found to have a significant correlation with poststroke function [12, 13, 24]. The third limitation was that our study was cross-sectional design, and there were no presentations that changes in knee strength over time were associated with present walking speed and VO_2_ peak. Finally, the sample size was relatively small. Even though our study was representative of the population in our center, larger or multicenter studies are suggested to be carried out to further research and confirm our results.

## 5. Conclusion

Walking speed and peak oxygen uptake are markedly impaired after stroke and they made greatest impact on stroke patients' daily life. Knee extensor strength, particularly 90-degree torque, has a good correlation to both walking speed and exercise capacity of community-dwelling stroke subjects. In regression analysis, the most powerful determinant of the exercise capacity and the walking speed was nonparetic and paretic knee extensor strength, respectively. Understanding these relationships can remind us that not only aerobic training but also strengthening exercise should be taken into account in designing an organized rehabilitation program for stroke patient and consequently promote them to return to independent community living.

## Figures and Tables

**Table 1:** Characteristics of participants (*N* = 35).

Age (years)	57.10 ± 12.14
Duration (years)	1.84 ± 1.86
Height (cm)	163.60 ± 10.69
Weight (kg)	68.93 ± 16.26
BMI (kg/m^2^)	25.60 ± 4.21
Sex	
Male	24 (68.6%)
Female	11 (31.4%)
Affected side	
Left	19 (54.3%)
Right	16 (45.7%)
Stroke type	
Infarctic	17 (48.6%)
Hemorrhagic	18 (51.4%)
Lesion location	
Main trunk	8 (22.9%)
Basal ganglion	12 (34.3%)
Brain stem	2 (5.7%)
Cortex	6 (17.1%)
Others	7 (20.0%)
Aid device	
Need	5 (14.3%)
No need	30 (85.7%)
Comorbidities	
Hypertension	28 (80.0%)
Dyslipidemia	16 (45.7%)
Diabetes	10 (28.6%)
Heart disease	7 (10.0%)
Renal disease	3 (8.7%)
Medication	
*β*-blocker	5 (14.3%)
Non-*β*-blocker	30 (85.7%)
Personal history	
Regular exercise habit	17 (48.6%)
Smoking	2 (5.7%)
Brunnstrom stage	upper lower
II	0 5
III	7 14
IV	24 10
V	4 6
ADL (*N* = 22)	
FIM	87.64 ± 14.14
FIM-motor	55.95 ± 12.15
FIM-cognition	31.68 ± 5.09
Spasticity	
Ashworth scale (low limb)	0.71 ± 0.75

ADL: activity of daily living, FIM: functional independent measurement FIM_motor: motor component of FIM, FIM_cognition: cognition component of FIM.

**Table 2:** Walking speed, peak torque of knee extensors, and exercise tolerance test results.

Walking speed (m/s)	Sp_12m	0.52 ± 0.28
	Sp_L6m	0.56 ± 0.31
	Sp_F6m	0.49 ± 0.25^a^

Knee extensor strength (torque, Nm)	60DT_PL	68.93 ± 40.44
	60DT_NPL	106.06 ± 42.82^b^
	90DT_PL	49.34 ± 26.84^d^
	90DT_NPL	70.55 ± 31.93^c,e^

Exercise capacity variables	VE (times/min)	37.27 ± 12.51
	VO_2_ peak (L/min)	1.21 ± 0.43
	VO_2_/Kg (mL/min/kg)	17.55 ± 5.57
	HRmax (beats/min)	122.43 ± 19.43

Sp_12m: speed of total 12 meters; Sp_L6m: speed of last 6 meters; Sp_F6m: speed of first 6 meters; 90DT_PL: 90-degree torque of paretic leg; 90DT_NPL: 90-degree torque of nonparetic leg; 60DT_PL: 60-degree torque of paretic leg; 60DT_NPL: 60-degree torque of nonparetic leg; VE: ventilation; VO_2_ peak: peak O_2_ consumption; HRmax: maximal heart rate.

^a^ *P* < 0.00 between Sp_L6m and Sp_F6m; ^b,c^ *P* < 0.00 between NPL and PL.

^d,e^ *P* < 0.00 between 60DT and 90DT.

**Table 3:** Pearson's correlation with walking speed and VO_2_ peak.

Variables	Sp_12m	VO_2_ peak
Duration	−0.067	0.002
Age	−0.220	−0.365∗
Height	0.236	0.487∗
Weight	0.357∗	0.584∗
BMI	0.259	0.396∗

FIM	0.464∗	0.430∗
FIM_motor	0.477∗	0.321
FIM_cognition	0.150	0.422

60DT_PL	0.619∗	0.535∗
60DT_NPL	0.448∗	0.479∗
90DT_PL	0.662∗	0.485∗
90DT_NPL	0.474∗	0.606∗
Sp_12m	—	0.465

VE	0.376∗	—
VO_2_ peak	0.465∗	—
HRmax	0.358∗	—

*P < 0.05.

FIM: functional independent measurement, FIM_motor: motor component of FIM, FIM_cognition: cognition component of FIM, Sp_12m: speed of total 12 meters; 90DT_PL: 90-degree torque of paretic leg; 90DT_NPL: 90-degree torque of nonparetic leg; 60DT_PL: 60-degree torque of paretic leg; 60DT_NPL: 60-degree torque of nonparetic leg; VE: ventilation; VO_2_ peak: peak O_2_ consumption; HRmax: maximal heart rate.

**Table 4:** Linear regression model for walking velocity and VO_2_ peak.

	Sp_12m			VO_2_ peak		
	Beta coefficient	*P* value	*R* ^ 2^	Beta coefficient	*P* value	*R* ^ 2^
90DT_PL	0.621	0.004	0.497	−0.124	0.614	0.422
90DT_NPL	−0.150	0.488		0.559	0.011	
FIM	0.153	0.280		0.107	0.707	
Sp_12m	—	—		0.242	0.214	
VO_2_ peak	0.211	0.214		—	—	

Sp_12m: speed of total 12 meters; VO_2_ peak: peak O_2_ consumption; 60DT_PL: 90-degree torque of paretic leg; 90DT_NPL: 90-degree torque of nonparetic leg, FIM: functional independent measurement.
